# Renal recipients’ knowledge and self-efficacy during first year after implementing an evidence based educational intervention as routine care at the transplantation clinic

**DOI:** 10.1186/s12882-021-02468-x

**Published:** 2021-07-15

**Authors:** Kristin Hjorthaug Urstad, Astrid Klopstad Wahl, Torbjørn Moum, Eivind Engebretsen, Marit Helen Andersen

**Affiliations:** 1 Faculty of Health Sciences, Department of Quality and Health Technology, University of Stavanger, 4036 Stavanger, Norway; 2 Faculty of Medicine, Department of Health Sciences, University of Oslo, Oslo, Norway; 3 Department of Transplantation Medicine, Oslo University Hospital, Oslo, Norway; 4 Faculty of Medicine, Department of Behavioural Sciences in Medicine, University of Oslo, Oslo, Norway

**Keywords:** Implementation, Patient education, Renal transplantation

## Abstract

**Background:**

Following an implementation plan based on dynamic dialogue between researchers and clinicians, this study implemented an evidence-based patient education program (tested in an RCT) into routine care at a clinical transplant center. The aim of this study was to investigate renal recipients’ knowledge and self-efficacy during first year the after the intervention was provided in an everyday life setting.

**Methods:**

The study has a longitudinal design. The sample consisted of 196 renal recipients. Measurement points were 5 days (baseline), 2 months (T1), 6 months (T2), and one-year post transplantation (T3). Outcome measures were post-transplant knowledge, self-efficacy, and self-perceived general health.

**Results:**

No statistically significant changes were found from baseline to T1, T2, and T3. Participants’ levels of knowledge and self-efficacy were high prior to the education program and did not change throughout the first year post transplantation.

**Conclusion:**

Renal recipients self-efficacy and insight in post-transplant aspects seem to be more robust when admitted to the hospital for transplantation compared to baseline observations in the RCT study. This may explain why the implemented educational intervention did not lead to the same positive increase in outcome measures as in the RCT. This study supports that replicating clinical interventions in real-life settings may provide different results compared to results from RCT’s. In order to gain a complete picture of the impacts of an implemented intervention, it is vital also to evaluate results after implementing findings from RCT-studies into everyday practice.

## Introduction

In recent years, considerable improvements have been made in the field of transplantation. However, shorter hospital stays combined with fewer follow-up appointments may have imposed greater demands on kidney recipients in terms of coping with life after transplantation [1–5]. Providing updated, effective patient education programmes for renal transplants is essential: the consequences of a lack of knowledge can be fatal (e.g. disregarding signs of rejection), and limited health literacy is recognized as a central barrier to successful transplantation [6, 7].

A randomized trial performed at a Norwegian hospital in 2012 tested the effect of a new education programme that emphasized individualizing and tailoring the educational content to each patient’s needs [8]. The programme significantly increased renal transplant recipients’ knowledge about post-transplant aspects and enhanced their self-efficacy, which are important predictors of health-related behaviour outcomes [9]. Following the positive results from this clinical trial and supported by other reports of the beneficial effects of tailored patient education programmes [10–15], we implemented the new education programme as routine practice at our transplant centre [16].

Health-care professionals have shown increased interest in facilitating the implementation of research results from academic settings to clinical practice. Such interest has been described using various concepts and with models using various names [17, 18]. Yet, there is still no consensus or clear guidance on how to implement results from controlled trials in real-world clinical health education settings to ensure the desired effects. It has been claimed that strong fidelity to the original intervention boosts effectiveness in terms of attaining intended intervention goals [19–22]. However, implementation is a complex process. According to Greenhalgh and Wieringa [23], an intervention developed in an experimental context cannot unequivocally be transferred to a real-world setting without contextual adaption. Thus, the implementation at the transplant centre in the present study was based on viewing implementation as a bidirectional process (between researchers and clinicians) that depends on dynamic dialogue and allows for local adjustments [24–26].

This study is the fourth in a row within a broader implementation project aiming to examine the implementation of the new education program, from different angles and in different phases utilizing different study designs [16, 27–29]. The focus of the current study was whether the positive effects of the randomized controlled trial (RCT) intervention could be recaptured in a long-term perspective. Hence, the specific aim of this was to investigate renal recipients’ knowledge and self-efficacy during first year the after the intervention was provided in an everyday life setting.

### Background

#### Clinical Context and the Evidence-Based Patient Education Programme tested in the RCT study

The study was conducted at the only transplant centre in Norway. Annually, it performs about 270 kidney transplants and employs approximately 100 nurses within a surgical unit, medical unit, and outpatient clinic. Patients are discharged from the hospital approximately one week post-transplantation. After discharge, patients attend the outpatient department unit medical controls for about 8–10 weeks. Except for patients living close to the centre, renal transplant recipients stay at the patient hotel situated close to the hospital.

The main differences between conventional patient education used at the transplant centre and the evidence-based patient education programme were the emphasis on individual adaptation, the method of knowledge transfer, the later timing of the education program and the extended number of training sessions.

To ensure individualisation, the evidence-based patient education programme utilized the Academic Detailing method [30]. This method comprised the following aspects: identification of baseline knowledge and needs (screened by posing knowledge questions to patients); defining evident training areas; using skilled instructors; encouraging active patient participation; repeating and elucidating key areas; and providing feedback about behaviour change [30]. The Academic Detailing method is based on social cognitive theory, which regards human behaviour as a product of the dynamic interplay among personal, behavioural, and environmental influences [31].

Previously at the centre, patient education had been provided during the 7–10 days of hospitalization while the new evidence-based patient education programme was provided during 7 weeks of post-transplant outpatient care. The programme consisted of five one-to-one educational sessions and main teaching focus were immunosuppressive medication, organ rejection, and lifestyle. With each of those themes, content was contextualized and adapted based on each patient’s needs. Each session lasted from 40 to 60 min and was provided by the research nurse in the RCT study.

#### Patient education history at the study transplant centre

Some events during the last years should be made explicit at they might have impacted on the patient education situation in our study context. Since the RCT study was performed in 2012, the standard written information previous handed out to the patients when attending to the hospital has been digitalized and made available online. Another factor related to dissemination of information was that the transplant centre had participated in a documentary program shown on one of the main Norwegian TV channels. In this documentary of 10 episodes, patients shared their experiences of living with kidney disease and going through a transplantation.

Routines for follow-up care and the structure and content of the education program for renal transplant recipients were comparable to the current study.

#### Implementation

The implementation of the new education program was inspired by the Formative Evaluation Consultation And Systems Technique (FORECAST) approach which emphasizes collaboration and mutual influence between the project stakeholders and research teams; it provides advice for continuous evaluation throughout the implementation [32]. We established an implementation team comprising researchers who were involved in the original RCT reference study [8] nursing staff from the three transplant units, and a patient representative. In-depth discussions about the new programme and feedback loops helped identify and accommodate the possible need for ongoing changes [32]. This resulted in the following agreed-upon changes: The number of sessions was reduced from five to three as that was believed to require fewer extra resources but was still regarded as sufficient to achieve the intervention goals. Further, the pen-and-paper response knowledge screening was changed to patient-nurse face-to-face screening. All the main topics remained the same. However, it was agreed to focus less on self-monitoring (such as measuring urine protein) and pain killer advice. According to updated evidence-based practice these areas were regarded as less relevant for the patient education content.

In order to promote mutual understanding of the new patient education programme the implementation team developed educational sessions for the transplant nurses that would be providing the new education program to the patients. This included an introductory lecture concerning pedagogical theory related to tailored education and the aspects of the academic detailing tailoring method [30]. Furthermore, different patient education scenarios were discussed first in small groups and in plenary during a workshop session. The ½ day course was delivered at three different times to ensure that most nurses could attend the course, due to variations in their shift work schedules.

## Methods

This study has longitudinal design. As with the reference study, the primary outcome was post-transplant knowledge [4]; the secondary outcome was self-efficacy [33].

Our measurement points were at baseline (5 days post-transplantation), T1 (2 months post-transplantation), and T2 (6 months post-transplantation). All measure points in the current study were in accordance with the measurement points in the reference study. In both study settings, standard care of patients was treatment in hospital during the first week after the kidney transplantation. The standard follow-up program for the patients at the transplant centre had not changed since the RCT study was conducted, therefor the measure point at baseline was assumed to be comparable to the reference RCT study. To capture longer-term changes, the present study included in addition a measurement point 1 year post-transplantation (T3).

### Ethics

 The study was approved by the Data Protection Office at Oslo University Hospital (2014/5573). Written informed consent was obtained from each participant prior to study procedures. All participants were informed that their data would be anonymous and treated with confidentiality. They were informed about publication plans. They were told they could withdraw from the study at any time without any consequences to them.

### Recruitment and Sample

Participants were recruited by trained nursing staff at transplant centre in Norway within the first days after kidney transplantation. The criteria for inclusion were being above 18 years of age, being able to participate in post-transplantation patient education, and being able to read Norwegian sufficiently to complete the questionnaire. Based om the sample size calculation from the reference study, it was estimated that a sample of around 200 was required.

Among the 357 patients who received kidney transplants between February 2016 and August 2017, 111 were for administrative reasons (holidays or busy days at the transplant clinic) not invited; 29 did not meet the inclusion criteria (25 were unable to read Norwegian). Of the 217 patients asked to participate in the study by trained nursing staff, 199 agreed. Of those 199, three were excluded (two patients because they did not follow the patient education; one had been erroneously included). Ultimately, 196 patients were included.

### Measures

As with the reference study, we applied the Knowledge Questionnaire for Renal Recipients [4] and we measured self-efficacy using the General Self-Efficacy Scale [33]. The reference study also measured health-related quality of life. However, as no effect in total health-related quality-of-life scores was observed; the present follow-up study only used a single item to assess self-perceived general health.

The Knowledge Questionnaire for Renal Recipients was developed in Norway; it focuses on medication, rejection symptoms, and lifestyle [4]. Owing to changes in patient education content, we revised that used in the reference study in 2007–2009: we deleted five irrelevant items. The questionnaire employed in the present study contained 14 items. The five-point response scales ranged from ‘totally disagree’ to ‘totally agree’. When scoring the questionnaire, we accorded points only to completely correct answers (i.e. 1 or 5); we summed the total score of correct answers. The total possible score for the questionnaire thus ranged from 0 to 14, with higher scores indicating better knowledge.

The General Self-Efficacy Scale (GSE) contains 10 statements reflecting an individual’s belief in their ability to respond to novel or difficult situations [33]. Each statement has a four-point response scale, ranging from ‘not at all true’ to ‘completely true’. The GSE score totals range from 10 to 40 points, with a higher score indicating better self-efficacy. The questionnaire has been translated into Norwegian, and it shows satisfactory reliability and validity [34].

The single item to assess self-perceived evaluation of total health was posed as follows: ‘In general how would you rate your health?’ The response categories were ‘excellent’, ‘very good’, ‘good’, ‘fair’, and ‘poor`. A score above 5 indicates lower evaluation of health [35]. The question has been translated into Norwegian and validated [36].

### Analysis

We used IBM SPSS® statistics for Windows version 25 (IBM Armonk, NY)) for the statistical analyses. We performed a paired sample *t* test for each outcome measure from baseline to T1, T2, and T3. We investigated statistically significant differences in levels of knowledge in terms of gender, age, educational level, and duration of kidney disease using univariate regression analysis.

## Results

### Sample Characteristics

Most respondents were men (67.7 %) and living with someone (73.5 %). Their mean age was 56 years (range, 20–81 years). Before transplantation, 50 % received social security, 36.6 % were employed, and 13.5 % were students, unemployed, or full-time home-maker. Almost one-fifth (19 %) of participants reported having completed over 4 years of university or college education. The available baseline demographics for the participants in the implementation study and those in the reference study appear in Table 1.

**Table 1:** Demographics for participants in the implementation study and for the participants in the reference RCT study

	Participants in the implementation study			Participants in the reference RCT study		
Variable	Total n	n (%)	Mean (SD) range	Total n	n (%)	Mean (SD) range
Age	199		56 (14) (20–81)	159		51 (14) (21–77)
Gender	196					
Male		133 (67.7)			110 (69.1)	
Female		63 (32.3)			49 (30.9)	
Marital status	189			159		
Married/living with a partner		139 (73.5)			96 (60.3)	
Single, divorced, separated		50 (26.5)			63 (39.6)	
Education	172			159		
Completed high school (10–13)		158 (91.8)			132(83)	
Completed higher education, less than 4 years		57 (33.1)			61 (38.3)	
Completed higher education, over 4 years		33 (19.1)			30 (19)	
Employment status	172			159		
Paying job		63 (36.6)			78 (49)	
Disability insurance or retirement pension		86 (50)			69 (43.3)	
Other (student, unemployed, full-time householder)		23 (13.5)			12 (7.5)	
Length of time living in Norway (years)	168		54 (16) (5–78)		*Not measured*	
Norwegian language skills:	165				*Not measured*	
Excellent		128 (77.6)				
Very good		21 (12.7)				
Good		15 (19.1)				
Farley good		1 (0.6)				

We categorized the underlying kidney disease according to the annual report of the US Scientific Registry of Transplant Recipients and the Organ Procurement and Transplantation Network [37]. The proportion of living-donor transplants was lower for participants in the implementation study compared to participants in the reference study (27.7 % versus 48 %, respectively) and post-operative complications were less prevalent (9.8 % versus 18 %, respectively). Clinical characteristics for participants in the implementation study and in the reference RCT study are presented in Table 2.

**Table 2:** Clincal characteristics of participants in participants in the implementasjon study and in the reference RCT study

	Participants in the implementation study			Participiants in in the reference RCT study	
Variable	Total n	n (%)	Mean (SD) Median (range)	Total n	n (%)
**Kidney disease**	172			159	
Glomerular disease		40 (23.6)			56 (35)
Diabetic nephropathy		19 (11.2)			16 (11)
Hypertensive nephrosclerosis		26 (15.3)			24 (15)
Polycystic kidney disease		23 (13.6)			25 (16)
Renovascular/other vascular		6 (3.5)			8 (5)
Other		32 (18.9)			30 (19)
Do not know kidney disease diagnosis		25 (13.)			0
Previous kidney transplant	191	35 (18.3)		159	23(14)
Kidney from living donor	191	52 (27.2)		159	77 (48)
Post-transplant complications	164	16 (9.8)		159	30 (18)
Duration of kidney disease	166		16.4(13.8) 11(1–55)	156	Duration of disease prior to Tx n (%): Less than 2 year: 20 (13) Between 2 and 10 year: 64 (41) More than 10 year: 72 (46)

### Outcome Measures

We observed no statistically significant changes in levels of post-transplant knowledge (primary outcome) or self-efficacy (secondary outcome) from baseline to 7 weeks, 6 months, or 1 year post-transplantation. Furthermore, we found no statistically significant differences in post-transplant knowledge according to gender, age, education level, or duration of kidney disease at any of the measurement points. The overall health decreased significantly from baseline to 7 weeks post-transplantation; however, no significant changes were evident from baseline to 6 months or 1 year post-transplantation. The descriptive information and paired sample *t* test results for each outcome measure from baseline to T1, T2, and T3 are presented in Table 3.

**Table 3:** Descriptive information and details of paired sample t-tests for each outcome measures from baseline to T1, T2 and T3

Instruments and scoring range	Baseline	T1	T2	T3	Baseline -T1			Baseline – T2			Baseline – T3		
	Mean (N) SD	Mean (N) SD	Mean (N) SD	Mean (N) SD	Effect Size	( CI )	*P*- value	Effect size	(CI)	*P*-value	Effect Size	(CI)	*P*- value
Transplantation knowledge questionnaire (0–14)	10.04 (171) 2.59	10.29 (157) 2.54	9.92 (162) 2.76	9.86 (160) 2.66	-1.02 (-0.58-1.18) 0.39			0.78 (-0.24-0.54) 0.43			0.91 (-2.2-0-59) 0.37		
General Perceived Self-Efficacy Scale (GSE) (14–36)	32.32 (162) 5.0	32.53 (154) 4.59	32.14 (161) 5.76	31.83 (160) 4.86	-1.57 (-1.11-1.12) 0.37			-0.91 (-1.31-0.48) 0.37			0.93 (-0.57-1.58) 0.36		
Overall health (1–5)	2.87 (170) 0.93	2.61 (157) 0.88	2.76 (160) 0.86	2.72 (158) 0.87	4.48 (0.18–0.47) 0.00*			0.46 (-0.12-0.18) 0.64			1.92 (-0.0-0.30) 0.56		

*Statistically significant (*p*-value <0.05%)

At baseline, the mean for correct post-transplant knowledge answers in the experimental group in the reference study was 58.5 % (n = 78); mean for correct post-transplant knowledge answers in the implementation study was 72.1 % (*n* = 171). The scores for the knowledge questionnaire in both study populations at baseline appear in Table 4.

**Table 4:** Baseline scores for the Knowledge Questionnaire for Renal Recipients for participants in the reference and implementation study

Knowledge Questionnaire for Renal Recipients	Participants in the reference RCT study	Participants in the implementation study
**Items related to medication**:	Correct answer n (%)	Correct answer n (%)
Item 1: *If I stop taking immunosuppressive medication, my kidney will no longer function.*	139 (88)	143 (84)
Item 2: *It is not important to take immunosuppressive medication morning and evening as long as the total amount for the day is taken*.	123 (78)	148 (87)
Item 3: *If I throw up my medication, I should contact the doctor.*	119 (75)	106 (62)
*Item 4: I should not drink grapefruit juice after my kidney transplant.*	91 (58)	145 (85)
**Items related to rejection**:		
Item 5: *Rejection means that the body’s immune system attacks the kidney.*	142 (90)	153 (90)
Item 6: *If I have a rejection, I will no longer have a functional kidney.*	53 (34)	47 (28)
**Items related to lifestyle**:		
*Item 7: There is not much I can do to prevent the side effects of my medication.*	44 (28)	56 (33)
Item 8: *After the kidney transplant, my immune system is so weak that I should not travel by bus or other public transport.*	108 (68)	114 (67)
Item 9: *It is important to drink at least 2 L of fluid a day also after I have been discharged. from the hospital*	120 (75.9)	41 (83)
Item 10: *The medication gives me poor appetite, so I should eat food with high levels of calories.*	93 (59)	103 (61)
Item 11: *After the kidney transplant, I need to be careful to protect me from the sun.*	132 (83.5)	152 (90)
*Item 12: It is important to take it easy and avoid exercise during the 1st year after the kidney transplant*.	108 (68)	138 (81)
*Item 13: I have no possibilities to have more than one kidney transplant.*	131 (83)	145 (85)
*Item 14: The Kidney Patient Association can be a useful resource for me after my kidney transplant.*	102 (65)	125 (73)

At T1, participants in the experimental group in the reference study showed an increase in the mean for correct post-transplant knowledge answers from 58.5 to 65 % (*n* = 71). By contrast, the participants in the implementation study showed an increase only from 71 to 73.5 % (*n* = 157).

At T2, the experimental group in the reference study showed an increase in correct answers of up to 70 % (i = 64). Participants in the implementation study demonstrated a stable mean of 71 % correct answers (*n* = 160). Self-efficacy score in the experimental group at baseline was 28.7 (SD 3.7) and significantly increased to 30.8 (SD 4.2) after 7 weeks and 34 (SD 4.3) after 6 months. In the implementation study, self-efficacy score was 32.32 (SD) and did not significantly change throughout the first year after the transplantation.

## Discussion and conclusion

### Discussion

This study investigated whether the positive effect of an evidence-based educational intervention could be recaptured in a long-term perspective after being implemented in an everyday setting. The implementation process followed a structured plan: the present study employed the same measurement points and outcome instruments as the reference RCT study [8]. However, we did not observe the same changes as before. It is well recognized that replicating clinical interventions in real-life settings presents challenges [38]. To provide sustainable health-care services, there are calls for more implementation research aiming to better bridge research and practice [39]. Implementation science explores what we can learn from real-life tests, also when studies report non-significant results. By reflecting on possible explanatory factors, the present study should therefore be regarded as an important contribution for understanding processes of implementation in patient education contexts.

In this study, we found that patients’ levels of knowledge and self-efficacy were already high prior to the educational sessions (comparable to the post-intervention outcome levels in the reference study). One explanation for this could be the recent years advances in kidney transplantation treatment. Better surgical technics and post-operative anaesthetic management care might have led to a faster post-transplantation recovery for the current study population [40–43]. This is supported by the present study findings: fewer post-operative complications were evident in the current sample than in the reference study. Because of this, patients in the current study were perhaps more oriented towards their new situation at an earlier stage compared to the patients in the reference RCT study. As patients in the current study context started to recover from surgery, it is likely to believe that they also started to seek information through bedside interaction with transplant health personnel. Therefore, they might have gained more insight into post-transplant aspects at 5 days post-transplantation (baseline) before the education program started, compared to the patients in the reference RCT study. This is positive for transplant care as patient education potentially can start at an earlier stage than before. However, this makes it challenging to compare the two study populations at baseline. Using similar measure points in replicating RCT studies is highly recommended [44]. However, our findings illustrate how rapid progress in treatment and heath can makes this complicated.

A more general factor explaining the difference in post-transplantation findings between the reference and implementation study groups could be the shift in health education needs. The rapid proliferation of and access to health information on the Internet have created new possibilities related to health information seeking. Patients have become increasingly comfortable using the Internet as a prime source of basic health information. In the context of transplantation, learning material has been made available online and through TV programs. Through social network platforms such as Facebook, patients are increasingly sharing their disease experiences [45]. Shared lived experiences are by patients valued as one of the most important sources of information and research indicates that Internet-informed patients are more active knowledge seekers and ask more questions of and request additional information from health personnel [45, 46]. Patients with chronic kidney disease often have a long history of health-care personnel interactions. Aided by available Internet health resources, they may have been able to discuss their health issues more actively with health-care personnel and, therefore, have been more prepared for the transplantation.

It is also necessary to reflect on the implementation process and the challenge of finding an appropriate balance between fidelity to the original intervention and adaptation [24]. The implementation process in the present was built on mutual dialog between researchers and clinicians and after agreed upon adjustments, the implemented programme was no longer identical to the “package” in the reference study. With an RCT design, high fidelity to the intervention is the gold standard [46]. However, in an implementation setting, adaptation of an intervention is the rule rather than the exception [47, 48]. In the current study, the most significant change from the original intervention, was perhaps that the number of sessions was reduced from five to three. It was through in-depth discussions between researchers and clinicians agreed that three sessions were sufficient to achieve the intervention goals. To provide cost-effective treatment is a continuing battle in health care. This factor might have impacted on this decision and there is a potential risk that reducing the session numbers might have impacted more on the intervention than assumed. Precisely how the local adjustments affected the intervention in the present implementation is difficult to define. Complex interventions in health care comprise a number of components that may act independently —although the ‘active’ ingredients may be difficult to specify [49].

Another aspects explaining a possible lack of intervention impact is that in the original RCT study, the educational intervention was provided by the research nurse while the implementation study it was provided by the transplant nurses. This might have made a difference in terms of commitment to the intervention and consistency of deliver. On the other hand, a survey of transplant nurses perceptions of the implemented education program demonstrated that they felt confident and had enough knowledge about the new program and were motivated for using it in their nursing practices [29].

This follow-up study is the fourth in a series that presents results from within a broader implementation project, which follows the implementation of a new education programme for kidney recipients [16, 27–29]. It is important that the present results are understood with respect to results from the other follow-up studies. In the survey of transplant nurses’ perception, the nurses reported that the new program provided a holistic educational approach and that patients with special educational needs were better taken care of [29]. These findings were supported from an ethnographic observation study that analysed 19 of the teaching sessions in the new program [27]. This study revealed a wider patient-tailoring approach than the original intervention’s mapping of patient knowledge because the nurses also actively engaged with the patient’s life world. Furthermore, qualitative interviews with kidney transplants indicated that the new education program provided contextualized and individualized knowledge that were valuable for everyday life [28]. Cleary, the new patient education program represented a positive change. An important question is therefore whether the outcome measures used in the current study were suitable. As patients were more competent at the start of the education program, the tailored education content was most likely more advanced in the current study compared to reference study. Consequently, the instrument from the controlled RCT might not be suitable as the 19 items in the knowledge questionnaire measuring basic transplant knowledge were might not sensitive enough to capture patients` new insight. This demonstrates the importance of examine the implementation from different angles and with different methods.

Another interesting finding to discuss why the patients did not get more knowledgeable confident over time. This is surprising, as one could have expected that life experience throughout the first-year post-transplant would have led to higher competences. Other studies have found that the effect of patient education wanes over time as distance from the intervention widens [50]. If this is the case in our study is difficult to say, as we did not see any changes from baseline at all. Again, the outcome instruments measuring basic knowledge, might be the best explanation for the lack of observed increase in patients transplant knowledge.

A limitation of this study is the large number of patients who were not approached for participation, which was due to less focus on including patients during busy periods and holidays. Due to ethical constrains, we were unable to gather information about participants who were not included in the sample; thus, we do not know if they deviated considerably from the study sample. However, except for the lower number of post-operative complications and less living donor transplant, the demographical and clinical characteristics of participants in the implementation study were comparable to the sample in the reference RCT study.

### Conclusions

In conclusion, there are several possible explanations for why we did not observe the same changes in the implementation study as with the reference RCT. This study highlights the complexity of determining the success of educational programmes in a shifting clinical setting. Renal recipients’ self-efficacy and insight in post-transplant aspects seem to be more robust when admitted to the hospital for transplantation and this may be one of the explanation of why the implemented educational intervention did not lead to the same positive increase in outcome measures as in the RCT. This study supports that replicating clinical interventions in real-life settings may provide different results compared to results from RCT’s. In order to gain a complete picture of the impacts of an implemented intervention, it is vital also to evaluate results after implementing findings from RCT-studies into everyday practice.

### Practical Implication

Future efforts should focus on obtaining insights into implementation and post-implementation outcomes: this study has demonstrated that evidence-based educational interventions may not always work as expected in a real-life setting. To gain a more complete picture of the impact of an implemented intervention, it will be necessary to apply complementary study designs.

## Data Availability

Data is available on request to the corresponding author.
